# Effects of penehyclidine hydrochloride combined with dexmedetomidine on pulmonary function in patients undergoing heart valve surgery: a double-blind, randomized trial

**DOI:** 10.1186/s12871-023-02176-z

**Published:** 2023-07-13

**Authors:** Fang He, Yizhi Lu, Qi Mao, Lifang Zhou, Yanhua Chen, Yubo Xie

**Affiliations:** 1 Department of Anesthesiology, The First Affiliated Hospital of Guangxi Medical University, Nanning, China; 2 Guangxi Key Laboratory of Enhanced Recovery After Surgery for Gastrointestinal Cancer, The First Affiliated Hospital of Guangxi Medical University, Nanning, China

**Keywords:** Penehyclidine hydrochloride, Dexmedetomidine, Cardiopulmonary bypass, Heart value surgery, Pulmonary function

## Abstract

**Aim:**

To investigate the effects of penehyclidine hydrochloride combined with dexmedetomidine on pulmonary function in patients undergoing heart valve surgery with cardiopulmonary bypass (CPB).

**Methods:**

A total of 180 patients undergoing elective heart valve surgery with CPB were randomly divided into four groups: 45 in group P (intravenous penehyclidine hydrochloride 0.02 mg/kg 10 min before anesthesia induction and at the beginning of CPB, total 0.04 mg/kg); 43 in group D (dexmedetomidine 0.5 μg/kg/h after induction of anesthesia until the end of anesthesia); 44 in group PD ( penehyclidine hydrochloride 0.04 mg/kg combined with dexmedetomidine 0.5 μg/kg/h intravenously during anesthesia); and 43 in group C (same amount of normal saline 10 min before and after anesthesia induction, to the end of anesthesia, and at the beginning of CPB). The main outcomes were the incidence and severity of postoperative pulmonary complications (PPCs). The secondary outcomes were: (1) extubation time, length of stay in intensive care, and postoperative hospital stay, and adverse events; and (2) pulmonary function evaluation indices (oxygenation index and respiratory index) and plasma inflammatory factor concentrations (tumor necrosis factor-α, interleukin-6, C-reactive protein and procalcitonin) during the perioperative period.

**Results:**

The incidence of PPCs in groups P, D and PD after CPB was lower than that in group C (*P* < 0.05), and the incidence in group PD was significantly lower than that in groups P and D (*P* < 0.05). The scores for PPCs in groups P, D and PD were lower than those in group C (*P* < 0.05).

**Conclusion:**

Combined use of penehyclidine hydrochloride and dexmedetomidine during anesthesia reduced the occurrence of postoperative pulmonary dysfunction, and improved the prognosis of patients undergoing heart valve surgery with CPB.

**Trial registration:**

The trial was registered in the Chinese Clinical Trial Registry on 3/11/2020 (Registration No.: ChiCTR2000039610).

**Supplementary Information:**

The online version contains supplementary material available at 10.1186/s12871-023-02176-z.

## Background

There are some serious complications after cardiopulmonary bypass (CPB) in patients undergoing cardiac surgery. The lungs are sensitive and fragile organs that are susceptible to ischemia/reperfusion injury and systemic inflammatory response [1, 2]. Patients after cardiac surgery often have different degrees of pulmonary dysfunction, which may be related to surgery, anesthesia and CPB, pulmonary ischemia/reperfusion, prolonged mechanical ventilation, inflammatory response, and protamine use [3]. Pulmonary dysfunction can increase postoperative morbidity, mortality, length of hospital stay, and healthcare burden [4].

Penehyclidine hydrochloride is a new type of long-acting selective cholinergic blocker with antimuscarinic and antinicotinic activities. It exerts its anticholinergic activity by selectively acting on M1 and M3 receptors distributed in the alveolar walls, airway smooth muscle, and submucosal glands. It can relax airway smooth muscle, dilate bronchioles, reduce mucus secretion and airway resistance, increase lung compliance, and improve respiratory function [5].

Dexmedetomidine is an α2 adrenergic agonist that selectively acts on parts of the brain or spinal cord. It can promote sedation and analgesia, and is widely used in anesthesia, procedural sedation, and sedation in intensive care [6]. In the clinical practice of one lung ventilation, dexmedetomidine has a protective effect on the lung by indirectly reducing the dose of other anesthetics that can have adverse effects on ischemic pulmonary vasoconstriction, or directly by reducing oxidative stress and increasing NO release [7], thereby enhancing hypoxic pulmonary vasoconstriction and improving oxygenation.

Penehyclidine hydrochloride and dexmedetomidine are commonly used as auxiliary anesthetics, and both have possible organ protective activity when the body is injured or under pathological conditions. However, the effects of combined use of penehyclidine hydrochloride and dexmedetomidine on patients undergoing cardiac surgery with CPB have not been studied. The current study investigated whether penehyclidine hydrochloride combined with dexmedetomidine had a positive effect on pulmonary function of patients undergoing heart valve surgery with CPB.

## Materials and methods

The study was approved by the Ethics Committee of the First Affiliated Hospital of Guangxi Medical University [Ethics No. 2020 No. (028)] and registered in the Chinese Clinical Trial Registry on 3/11/2020 (Registration No.: ChiCTR2000039610). Before the study, the patients were informed orally and in writing of the relevant details and risks, and gave signed informed consent. All methods were performed in accordance with the Declaration of Helsinki and relevant guidelines and regulations.

### Experimental design and case selection

We recruited 180 patients who underwent single/double heart valve replacement or valvuloplasty with CPB in the First Affiliated Hospital of Guangxi Medical University between May 2020 and September 2021. All patients were enrolled after screening according to the inclusion and exclusion criteria. The inclusion criteria were: (1) age 20–65 years, single/double heart valve replacement or valvuloplasty with CPB, weight 40–66 kg (BMI 18.5–23.9); and (2) ASA grade II/III and cardiac function NYHA grade II/III. The exclusion criteria were: (1) glaucoma; (2) prostatic hypertrophy; (3) poor preoperative pulmonary oxygenation, pulse oximetry of air inhalation < 90%, and pulse oximetry of pure oxygen inhalation < 95%; (4))emergency operation or secondary heart valve operation; (5) acute or chronic infectious diseases; (6) taking anti-inflammatory drugs and immunosuppressants; (7) lung, brain and other organ diseases or severe liver and kidney diseases; and (8) mini-mental state examination (MMSE) ≤ 23, severe vision or hearing impairment, illiteracy and/or communication difficulties related to pronunciation or dialect (involving another primary outcome in our study not elaborated in this article). The withdrawal criteria were: (1) those who reneged on participating in the trial and withdrew from the trial; (2) patients who voluntarily terminated treatment and were discharged during the trial; (3)patients with operation time > 8 h; (4) patients who died of nonrespiratory diseases during hospitalization; (5) patients with perioperative adverse events such as allergic reaction, cardiac arrest and stroke; (6) when the test process was likely to cause damage to the health or other rights of the subjects; and (7) termination of the trial was considered necessary from other medical perspectives.

### Randomization and grouping

Patients, data collectors, sample collectors and data analysts were not aware of the grouping. The patients were randomly divided into four groups with the help of Excel. The medication regimen of each patient was saved in an opaque envelope marked with the corresponding number by the research assistant who was not involved in data collection and anesthesia management. On the day of the operation, the assistant opened the envelope and filled the syringe with the drug or the same amount of physiological saline. Although the drug and physiological saline were transparent, they were still covered with an opaque label. According to the grouping, the corresponding drug/physiological saline was injected or continuously pumped at the corresponding time (10 min before anesthesia induction, after induction to the end of the operation, and at the beginning of CPB). Preoperative and postoperative assessments, perioperative data collection, and blood sampling were performed by another assistant who was not aware of the allocation scheme and was not involved in anesthesia management. The data were submitted to statisticians who did not participate in the experiment.

The patients were randomly divided into the following four groups: (1) penehyclidine hydrochloride group (group P): intravenous injection of 0.02 mg/kg of penehyclidine hydrochloride 10 min before anesthesia induction and at the beginning of CPB, with a total dose of 0.04 mg/kg; (2) dexmedetomidine group (group D): after induction of anesthesia, dexmedetomidine 0.5 μg/kg/h was pumped intravenously until the end of anesthesia; (3) penehyclidine hydrochloride plus dexmedetomidine group (group PD): penehyclidine hydrochloride 0.02 mg/kg was injected intravenously 10 min before anesthesia induction and at the beginning of CPB, with a total dose of 0.04 mg/kg; and dexmedetomidine 0.5 μg/kg/h was pumped intravenously after induction of anesthesia until the end of anesthesia; and (4) control group (group C): the same amount of normal saline was given 10 min before and after anesthesia induction to the end of anesthesia, and at the beginning of CPB.

### Anesthesia and CPB management

All patients were treated using the same anesthesia and perioperative management protocol. Routine prophylactic antibiotics (mostly cefuroxime 1.0 g) were used from 30 min to 1 h before the operation. When the operation time exceeded 3 h, the drugs were administered again after 3 h. There was no other preoperative medication. Cefuroxime (1.0 g) was used routinely after the operation, twice daily for two consecutive days. Antibiotics were changed or upgraded according to the change in disease condition. The selection and time of antibiotic treatment were based on the Guidelines for Clinical Use of Antibiotics (2015 version) issued by the National Health Commission.

After entering the operating room, a peripheral venous channel was established, and a five-lead electrocardiogram, pulse oximetry saturation, bispectral index (BIS), urine volume, and body temperature were monitored routinely. Left radial arterial puncture catheterization (20 G) and right internal jugular vein puncture catheterization (7F triple vena cava catheter) were performed under local anesthesia. The pressure sensors were connected to continuously monitor the invasive arterial pressure and central venous pressure.

Anesthesia induction: midazolam 0.05 mg/kg and etomidate 0.2–0.3 mg/kg were successively injected intravenously, followed by cisatracurium 0.3 mg/kg and fentanyl 10 μg/kg, and following intubation, mechanical ventilation was initiated. During anesthesia induction, norepinephrine and ephedrine were used to ensure sufficient perfusion pressure of important organs and maintain the stability of vital signs. Mechanical ventilation parameters were: tidal volume 8–10 ml/kg, ventilation frequency 10–12 times/min, inspiratory/expiratory ratio 1:2, air oxygen mixture flow 2 L/min, inspiratory oxygen concentration 60%, and end-expiratory CO_2_ partial pressure 35–45 mmHg.

Anesthesia maintenance: immediately after anesthesia induction, 1% propofol was pumped through the right internal jugular vein catheter at a constant rate of 2–8 mg/kg/h. The infusion rate of propofol was adjusted according to BIS to maintain the depth of anesthesia, so that BIS fluctuated within 40–55. During CPB, BIS was maintained at 30–40. During anesthesia, fentanyl 2 μg/kg was intermittently injected intravenously to maintain analgesia (total fentanyl 50 μg/kg), and cisatracurium 0.12 mg/kg/h was administered to maintain muscle relaxation. Dopamine, epinephrine, norepinephrine, and nitroglycerin micropump injection or small-dose single intravenous injection of vasoactive drugs such as norepinephrine, ephedrine and atropine, was used to maintain hemodynamic stability and maintain mean arterial pressure of 60–80 mmHg. At the end of anesthesia, infusion of all drugs was stopped except for continuous pumping of vasoactive drugs. The electrocardiogram, finger pulse oximetry monitoring and invasive blood pressure monitoring were transferred to the mobile monitor. After hemodynamic stability was assessed, the patients were escorted to the cardiothoracic surgery intensive care unit (ICU) together with the surgeon and operating room nurse.

Sodium lactate Ringer’s solution, hydroxyethyl starch electrolyte injection, 10% human albumin, 5% NaHCO_3_ and 2500 IU heparin were used as the priming solution for CPB. When activated clotting time (ACT) was ≥ 480 s, CPB was started. And ACT was maintained above 480 s during CPB. During CPB, rectal temperature was maintained at 31–32 °C. After CPB, the neutralizing and antagonistic dose of protamine was 1–1.5 times that of heparin, so that ACT reached the baseline level before heparinization. The perfusion flow of CPB was 2.4–2.8 L/min/m^2^, mean arterial pressure was 60–80 mmHg, and hematocrit was 22–28%. The strategy of α-stat was used in blood-gas management during CPB.

### Recovery management

All patients were transferred to the ICU after surgery, where they continued to receive respiratory and circulatory support. The mechanical ventilation mode was volume-controlled ventilation, and the fraction of inspired oxygen (FiO_2_) was 40–80%. Other parameters were adjusted by the ICU doctor according to the intraoperative conditions or blood gas analysis. Endotracheal tube was removed if the patients had stable respiratory and circulatory functions and clear consciousness; they could follow simple instructions (such as blinking, nodding and shaking hands); muscle strength was restored; and respiratory rate was 10 times/min, tidal volume continuously > 8 ml/kg, pulse oximetry saturation (SpO_2_) ≥ 95%, arterial oxygen saturation (SaO_2_) > 80 mmHg, and partial pressure of carbon dioxide in artery (PaCO_2_) < 50 mmHg. The patients continued oxygen inhalation after extubation and SpO_2_ was closely monitored.

### Outcome measures

The primary outcomes were the incidence and severity of postoperative pulmonary complications (PPCs). PPCs were diagnosed using the scoring standard that has been used and continuously optimized since 1992 [8–10]: Grade 0 (0 points) represented no symptoms or signs; Grade 1 (1 point) represented dry cough and microatelectasis (body temperature > 37.5 °C without other causes found in the lungs, and normal chest radiograph); Grade 2 (2 points) represented cough (many but not caused by other reasons), bronchospasm (new or pre-existing wheezing, requiring further treatment), hypoxemia (SpO_2_ ≤ 90% when breathing air), atelectasis (with radiological imaging evidence and temperature > 37.5℃ or abnormal lung findings), hypercapnia (PaCO_2_ > 50 mmHg); Grade 3 (3 points) represented the occurrence of pleural effusion (pleural puncture required), pneumonia (radiological evidence plus clinical symptoms or need to change antibiotics), pneumothorax, noninvasive ventilation, and secondary intubation after surgery (ventilator dependence ≤ 48 h); Grade 4 (4 points) represented respiratory failure (ventilator dependence > 48 h after surgery or secondary intubation); Grade 5 (5 points) represented death before discharge. The specific scoring criteria are shown in Table A1. Score ≥ 3 indicated the occurrence of PPCs.

**Table 1:** Baseline characteristics and surgical data

	**Group C (*n* = 43)**	**Group P (*n* = 45)**	**Group D (*n* = 43)**	**Group PD (*n* = 44)**	***P* value**
Gender (M/F), n	23/20	19/26	20/23	21/23	0.776
Age (yr)	48.4 ± 7.6	47.1 ± 9.9	50.2 ± 8.0	48.9 ± 6.6	0.350
Height (cm) (cm)	159.3 ± 6.8	158.2 ± 5.9	158.7 ± 6.5	158.1 ± 6.3	0.788
Weight (kg)	56.1 ± 8.3	56.9 ± 7.1	56.4 ± 6.2	55.4 ± 6.0	0.788
ASA					0.932
II, n (%)	17 (39.5)	20 (44.4)	20 (46.5)	19 (43.2)	
III, n (%)	26 (60.5)	25 (55.6)	23 (53.5)	25 (56.8)	
NYHA					0.973
II, n (%)	18 (41.9)	20 (44.4)	17 (39.5)	18 (40.9)	
III, n (%)	25 (58.1)	25 (55.6)	26 (60.5)	26 (59.1)	
Comorbidities n (%)					
Hypertension, n (%)	12 (36.4)	12 (26.7)	9 (20.0)	11 (25.0)	0.455
Diabetes, n (%)	8 (18.6)	7 (15.6)	6 (13.3)	7 (15.9)	0.919
Dyslipidemia, n (%)	9 (20.9)	10 (22.2)	7 (16.3)	8 (17.8)	0.897
Hepatic disease, n (%)	5 (11.6)	4 (8.9)	5 (11.6)	6 (13.6)	0.913
Smoking, n (%)	12 (27.9)	10 (22.2)	9 (20.9)	10 (22.7)	0.892
Preoperative PPC score	0.047 ± 0.21	0.067 ± 0.25	0.047 ± 0.21	0.046 ± 0.21	0.963
Duration of surgery (min)	297.4 ± 102.0	285.4 ± 84.2	304.6 ± 85.6	291.5 ± 79.8	0.754
Duration of anesthesia (min)	338.4 ± 99.6	327.4 ± 84.1	344.7 ± 85.4	331.4 ± 78.7	0.790
Duration of CPB (min)	134.5 ± 67.9	142.9 ± 69.7	131.4 ± 58.6	137.6 ± 64.1	0.860
Duration of ACC (min)	88.9 ± 54.9	93.6 ± 56.3	82.7 ± 47.5	83.9 ± 49.3	0.748
Ultrafiltration (ml)	2147 ± 680.4	2078 ± 654.6	2262 ± 711.5	2209 ± 695.4	0.385
Intraoperative infusion					
Allogeneic RBC (ml)	242 ± 139.3	237 ± 121.7	229 ± 129.2	234 ± 134.2	0.975
Plasma (ml)	388 ± 98.9	397 ± 106.2	378 ± 128.5	390 ± 141.0	0.907
Artificial colloid fluid (ml)	963 ± 424.7	934 ± 387.9	831 ± 376.2	889 ± 376.2	0.428
crystalloid fluid (ml)	1867 ± 565.1	1793 ± 533.2	1926 ± 507.6	1969 ± 550.2	0.452
urine volume (ml)	976 ± 446.2	852 ± 368.3	885 ± 341.3	897 ± 375.6	0.486
Cardiac resuscitation					0.542
automatically, n (%)	35 (81.4)	33 (73.3)	33 (76.7)	30 (68.2)	
Defibrillation, n (%)	8 (18.6)	12 (26.7)	10 (23.3)	14 (31.8)	
Surgical type, n (%)					
AVR, n (%)	12 (27.9)	9 (20.0)	8 (18.6)	9 (20.5)	0.745
MVR, n (%)	11 (25.6)	11 (24.4)	11 (25.6)	10 (22.7)	0.990
DVR, n (%)	20 (46.5)	25 (55.6)	24 (53.8)	25 (56.8)	0.760

The incidence and severity of PPCs were evaluated every day after surgery until discharge, and the highest score during hospitalization was the final score. The assessment and diagnosis were made by the ICU or respiratory physician, and the dates were recorded. The final scores and type of PPCs in each group were recorded.

The secondary outcomes were as follows. (1) Postoperative recovery time (time from the end of anesthesia to eye opening), extubation time (time from the end of anesthesia to the removal of the endotracheal tube), and length of ICU and postoperative hospital stay. (2) Pulmonary function evaluation indexes [alveolar arterial oxygen partial pressure difference (A-aDO_2_), oxygenation index (OI) and respiratory index (RI)] during the perioperative period: before anesthesia induction (T0), before skin incision (T1), before CPB (T2), 30 min after starting CPB (T3), 30 min after finishing CPB (T4), 6 h after finishing CPB (T5), and 24 h after finishing CPB (T6). (3) Adverse events (postoperative nausea, vomiting, abdominal distension, constipation, atrial fibrillation, heart failure, renal failure, ventricular fibrillation, and second admission to ICU). (4) Serum concentrations of tumor necrosis factor (TNF)-α and interleukin (IL)-6 were measured at T0, T2, T4, T5 and T6, and the concentrations of C-reactive protein (CRP) and procalcitonin were measured at T0, T4, T5 and T6.

### Statistical analysis and sample size calculation

IBM SPSS statistics 26.0 statistical software was used for data analysis. All measurement data were tested by normal distribution and variance homogeneity tests. One-way analysis of variance was used when the variance homogeneity test was passed. The results were expressed as means ± standard deviation. The least significant difference post hoc test was used for multiple comparisons of the four groups. When the variance homogeneity test showed that the variance was not uniform, Welch’s test was used for analysis, and Tamhane’s test was used for multiple comparisons between the post hoc test groups. Comparison between different time points was performed by repeated measurement analysis of variance. The numerical data were expressed as frequency and percentage, and analyzed by Fisher’s χ^2^ test. The results were expressed in percentages. The Bonferroni correction test was used for multiple comparisons of the four groups. The differences were considered statistically significant at *P* < 0.05. GraphPad Prism 6 software was used for statistical mapping.

The sample size was calculated on the basis of collecting the PPC scores for 40 patients in the four groups in the pretrial: PPC score was 2.2 points in the control group, 1.7 points in the penehyclidine hydrochloride group, 1.6 points in the dexmedetomidine group, and 1.4 points in the penehyclidine hydrochloride combined with dexmedetomidine group, α = 0.05, 1 − β = 0.80, the required sample size of each group (*n* = 39) was calculated using PASS 15.0 software. The patients could have withdrawn from the trial halfway through, or some data may have been missing, and there was a possible withdrawal rate of ~ 10%. After further calculation, 45 patients were required for each group, with a final estimate that 180 patients were needed.

## Results

### Baseline clinical characteristics

**Fig. 1:**
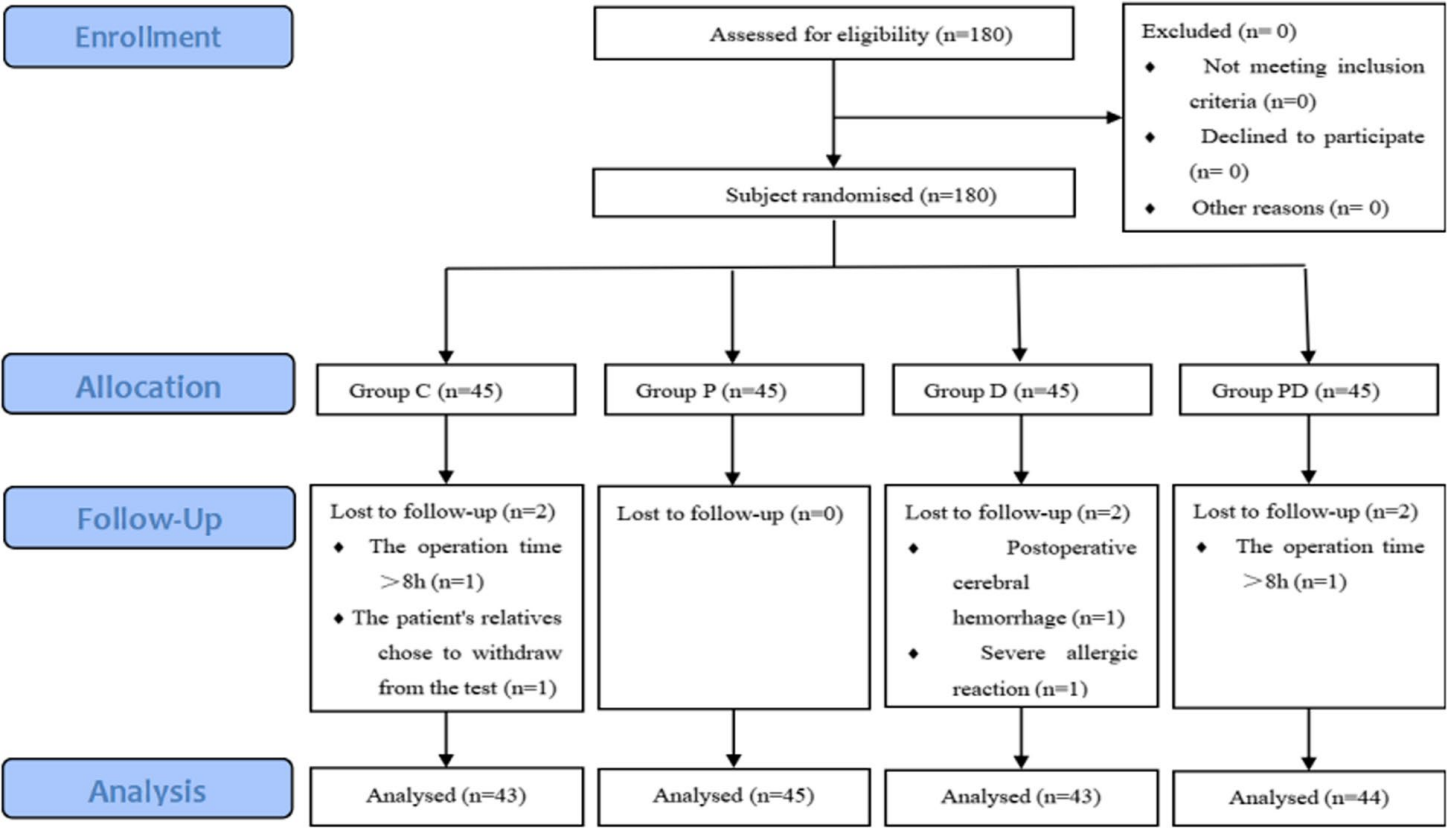
Patient randomization, follow-up, and analysis

The final analysis included 180 patients undergoing heart valve replacement, with 45 patients in each group. In Group C, one patient withdrew from the test because the operation time was > 8 h, and another patient’s relatives chose to terminate the test because of the low cardiac output after the operation; two patients withdrew from Group D because of postoperative cerebral hemorrhage or severe allergic reaction during the operation; and one patient withdrew from Group PD because the operating time was > 8 h. Therefore, 175 patients completed the study and were included in the data analyses (Fig. 1). There were no significant differences in baseline characteristics and intraoperative variables between the four groups (Table 1).

### Primary outcomes

The incidence of PPCs was 37.2% in group C, 17.8% in group P, 20.9% in group D, and 11.4% in group PD (*P* = 0.033) (Table 2). Compared with group C, the incidence of PPCs in group PD was significantly lower (*P* < 0.05), and the PPC score was significantly lower (2.07 vs 1.43, *P* = 0.001). However, there was no significant difference between groups P, D and PD in the PPC scores and incidence of PPCs (*P* > 0.05). Compared with group C, the proportion of patients with grade 1 PPCs in groups P and PD was significantly higher (*P* < 0.05), while the proportion of patients with other grade grades in each group showed no significant difference (*P* > 0.05).

**Table 2:** Comparison of PPCs among four groups

	**Group C** **(*n* = 43)**	**Group P (*n* = 45)**	**Group D (*n* = 43)**	**Group PD (*n* = 44)**	***P* value**
Postoperative PPC score	2.09 ± 1.04	1.53 ± 0.92^b^	1.60 ± 0.98^a^	1.43 ± 0.85^b^	0.007
PPCs incidence, n (%)	16 (37.2)	8 (17.8)	9 (20.9)	5 (11.4) ^a^	0.033
PPC score grading, n (%)					
0	3 (7.0)	3 (6.7)	4 (9.3)	3 (6.8)	0.960
1	9 (20.9)	24 (53.3) ^b^	19 (44.2)	25 (56.8) ^b^	0.003
2	15 (34.9)	10 (22.2)	11 (25.6)	11 (25.0)	0.573
3	13 (30.2)	7 (15.6)	7 (16.3)	4 (9.1)	0.070
4	3 (7.0)	1 (2.2)	2 (4.7)	1 (2.3)	0.572
PPCs type					
Pneumonia, n (%)	8 (18.6)	5 (11.1)	5 (11.6)	3 (6.8)	0.422
Pneumothorax, n (%)	3 (7.0)	0 (0.0)	1 (2.3)	1 (2.3)	0.207
Pleural effusion, n (%)	2 (4.7)	1 (2.2)	0 (0.0)	0 (0.0)	0.331
Secondary intubation, n (%)	0 (0.0)	1 (2.2)	1 (2.3)	0 (0.0)	0.870
Ventilator dependence > 48 h, n (%)	3 (7.0)	1 (2.2)	2 (4.7)	1 (2.3)	0.572

PPC scores after surgery were expressed as mean ± standard deviation. Incidence of PPCs, PPC scores and PPC types were expressed as cases of the number and percentage of patients [n (%)]. Compared with group C, ^a^*P* < 0.05 and ^b^*P* < 0.01

### Other outcomes

Compared with group C, the extubation time and length of ICU stay in group PD were decreased (*P* < 0.05) (Table 3). There was no significant difference in postoperative recovery time and length of hospital stay among the four groups (*P* > 0.05).

**Table 3:** Comparison of postoperative resuscitation among four groups

	**Group C** **(*n* = 43)**	**Group P (*n* = 45)**	**Group D (*n* = 43)**	**Group PD (*n* = 44)**	***P* value**
Wake-up time (h)	3.30 ± 1.18	3.50 ± 1.41	3.57 ± 1.46	3.59 ± 1.32	0.754
intubation time (h)	25.83 ± 14.2	20.79 ± 8.6	21.03 ± 12.4	18.5 ± 11.6^a^	0.035
Duration of ICU (h)	41.78 ± 19.2	35.31 ± 15.9	34.63 ± 16.7	30.94 ± 16.3^a^	0.031
Postoperative hospital stays (d)	6.28 ± 1.35	6.11 ± 1.27	6.21 ± 1.36	6.05 ± 1.20	0.841

Compared with group C, ^a^*P* < 0.05

OI of groups P, D and PD at T4, T5 and T6 after CPB was higher than that of group C (all *P* < 0.05) (Table 4). The OI of group PD 30 min after CPB (T4) was significantly better than that of groups P and D (*P* < 0.05).

**Table 4:** Comparison of oxygenation index among four groups

	**Group C** **(*n* = 43)**	**Group P (*n* = 45)**	**Group D (*n* = 43)**	**Group PD (*n* = 44)**	**statistic**	***P* value**
T0	460.8 ± 71.7	469.5 ± 85.7	453.3 ± 54.7	476.5 ± 72.8	1.032	0.382
T1	469.1 ± 73.5	486.3 ± 63.0	464.1 ± 53.5	487.9 ± 62.7	1.564	0.200
T2	470.3 ± 64.4	485.6 ± 48.3	468.0 ± 51.4	491.7 ± 36.8	2.602	0.057
T3	488.2 ± 68.8	483.4 ± 45.0	483.9 ± 60.6	480.1 ± 38.5	0.165	0.920
T4	298.5 ± 58.8^#^	346.3 ± 79.6^#b^	337.5 ± 73.3^#a^	378.0 ± 74.6^#bcd^	8.953	< 0.001
T5	328.1 ± 81.9^#^	387.6 ± 95.4^#b^	370.7 ± 75.6^#a^	398.6 ± 79.4^#b^	5.966	0.001
T6	346.5 ± 104.7^#^	401.3 ± 71.8^#b^	386.6 ± 95.8^#a^	411.0 ± 82.9^#b^	4.371	0.005

Compared with T0, ^#^*P* < 0.01; compared with group C, ^a^*P* < 0.05 and ^b^*P* < 0.01; compared with group P, ^c^*P* < 0.05; compared with group D, ^d^*P* < 0.05

RI of groups P, D and PD at T4, T5 and T6 after CPB was better than that of group C (*P* < 0.05) (Table 5). The RI of PD group 30 min after CPB (T4) was significantly better than that of groups P and D (*P* < 0.05).

**Table 5:** Comparison of RI among four groups

	**Group C** **(*n* = 43)**	**Group P (*n* = 45)**	**Group D (*n* = 43)**	**Group PD (*n* = 44)**	**statistic**	***P* value**
T0	0.37 ± 0.24	0.37 ± 0.28	0.39 ± 0.18	0.35 ± 0.21	0.218	0.884
T1	0.38 ± 0.26	0.32 ± 0.18	0.37 ± 0.17	0.32 ± 0.18	2.210	0.092
T2	0.36 ± 0.20	0.31 ± 0.14	0.37 ± 0.17	0.30 ± 0.10	2.050	0.109
T3	–	–	–	–		
T4	1.21 ± 0.53^#^	0.94 ± 0.59^#a^	0.96 ± 0.49^#a^	0.74 ± 0.33^#bcd^	6.620	< 0.001
T5	1.08 ± 0.73^#^	0.78 ± 0.64^#a^	0.78 ± 0.48^#a^	0.67 ± 0.44^#b^	3.999	0.009
T6	0.87 ± 0.93^#^	0.59 ± 0.39^#^	0.65 ± 0.74^#^	0.47 ± 0.50^#^	2.237	0.089

Compared with T0, ^#^*P* < 0.01; compared with group C, ^a^*P* < 0.05 and ^b^*P* < 0.01; compared with group P, ^c^*P* < 0.05; compared with group D, ^d^*P* < 0.05

**Fig. 2:**
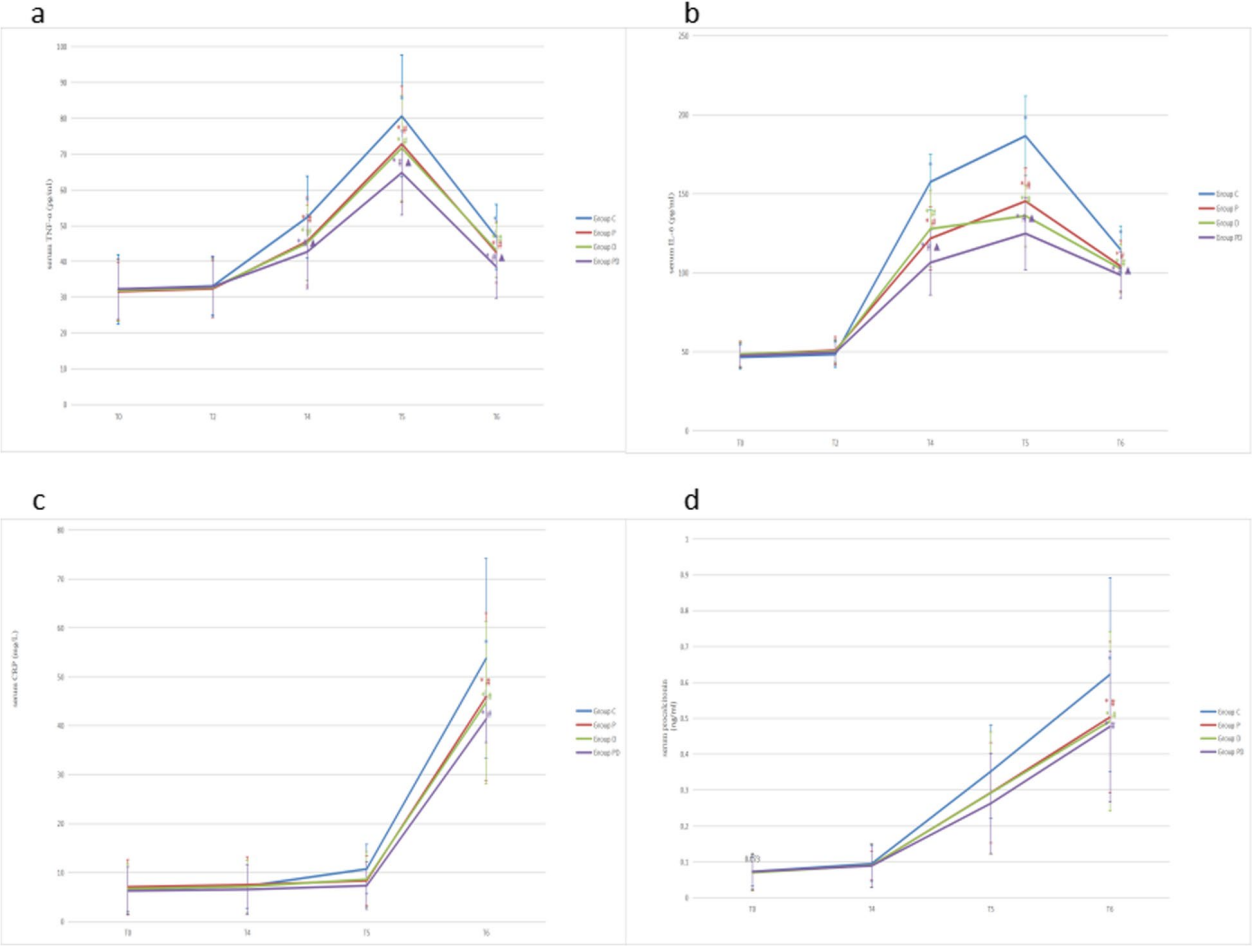
Comparison of serum TNF-α, IL-6, CRP and procalcitonin levels among four groups. The parameters depicted are **a** TNF-α, **b** IL-6, **c** CRP and **d** procalcitonin. ^*^P < 0.05 vs. T0, ^#^*P* < 0.05 vs. group C, ^▲^*P* < 0.05 vs. group P, D

To investigate whether administration of penehyclidine hydrochloride and dexmedetomidine during anesthesia for heart valve surgery reduced the release of serum inflammatory cytokines, we measured serum proinflammatory cytokine levels during and after surgery. Compared with before anesthesia (T0), the serum concentrations of TNF-α and IL-6 in the four groups at T4, T5 and T6 after CPB were significantly increased (*P* < 0.05) (Fig. 2a, b). Compared with group C, the serum concentrations of TNF-α and IL-6 in groups P, D and PD at T4, T5 and T6 were significantly decreased (*P* < 0.05). The serum concentrations of TNF-α and IL-6 in group PD were lower than those in groups P and D after CPB (*P* < 0.05). The serum concentrations of CRP and procalcitonin in the four groups were significantly increased at 24 h after CPB (T6, *P* < 0.05), and the serum concentrations of CRP and procalcitonin in groups P, D and PD were significantly lower than those in group C (*P* < 0.05) (Fig. 2c, d). There were no significant differences in serum CRP and procalcitonin concentrations among groups P, D and PD (*P* > 0.05).

## Discussion

This study showed that administration of penehyclidine hydrochloride and dexmedetomidine during anesthesia for cardiac valve surgery reduced the impact of CPB, cardiac surgery and anesthesia on lung damage, increased OI, and reduced release of inflammatory factors and the occurrence and severity of PPCs, thus improving the prognosis of patients. This is consistent with the previously reported protective effects of penehyclidine hydrochloride and dexmedetomidine on lungs during cardiac surgery or other pathological conditions [11, 12]. Our results showed that combination of penehyclidine hydrochloride and dexmedetomidine had a greater positive effect on the lungs than either of the two drugs alone.

Penehyclidine hydrochloride is a novel anticholinergic agent and previous studies have focused on treatment of organophosphorus poisoning and septic shock. Shen Weifeng et al. reported that penehyclidine hydrochloride pretreatment significantly reduced pulmonary histopathological changes, inflammatory cell infiltration and alveolar hemorrhage induced by lipopolysaccharide in rats [13]. Li Baiqiang et al. found that penehyclidine hydrochloride improved arterial partial pressure of oxygen in patients with acute lung injury (ALI) and prevented the development of ALI by downregulating expression of Toll-like receptor (TLR)4 and inhibiting inflammatory cytokines downstream of the TLR4 signaling pathway [14]. However, there have been few clinical studies on the organ protective activity of penehyclidine hydrochloride in adults undergoing cardiac surgery with CPB, and there is no uniform dose standard. Administration of penehyclidine hydrochloride 0.05 mg/kg before anesthesia induction reduces the incidence of ALI after aortic dissection [15]. In our study, we chose 0.04 mg/kg penehyclidine hydrochloride to study whether it combined with dexmedetomidine, in addition to being commonly used as auxiliary drugs for anesthesia, also had a positive impact on lung function. Dexmedetomidine is a clinically used sedative and analgesic. Continuous infusion of 0.5 μg/kg/h dexmedetomidine during anesthesia does not over-inhibit the cardiovascular system or cause hypertension, which meets the hemodynamic management requirements of cardiovascular anesthesia [16]. The lung protective effect of dexmedetomidine is mainly achieved by reducing inflammation and pulmonary edema [17], inhibiting oxidative stress [18] and reducing apoptosis of pulmonary tissue [19].

PPCs are the main cause of increased morbidity and mortality in patients undergoing surgical anesthesia [20]. The pathogenesis of PPCs is mainly atelectasis and restricted pulmonary ventilation, which eventually lead to respiratory disorders, and the causes of PPCs are related to the process of anesthesia, type of surgery, and patient-related risk factors [21]. General anesthesia affects respiratory movement and alveolar gas content by changing the diaphragm movement and chest size, while airway closure, reduction of pulmonary volume, stagnation of airway secretions, and mismatch of ventilation and blood flow perfusion are more obvious in thoracic surgery. Increased atelectasis and respiratory effort during anesthesia increase the time of postoperative mechanical ventilation and the risk of hospital-acquired pneumonia [22]. This means that even without pulmonary injury, mechanical ventilation itself may be a major determinant of PPC development. The CPB required for cardiac surgery and large area of atelectasis after thoracotomy can activate pulmonary inflammation, which magnifies the related harm of perioperative mechanical ventilation [10]. Therefore, general anesthesia and thoracotomy with CPB may impair respiratory function, with pulmonary complications such as hypoxemia, pneumonia, CPB-related pulmonary injury, and acute respiratory distress syndrome. During cardiac surgery, CPB, ascending aorta occlusion, vena cava occlusion and reoperation may lead to ischemia/reperfusion injury in the lungs, heart and other vital organs [23]. Thus, pulmonary ischemia/reperfusion injury and inflammation contribute to a higher incidence of PPCs after CPB compared with other types/sites of surgery.

In the current study, the PPC score of patients who used penehyclidine hydrochloride and dexmedetomidine was lower than that of patients who did not use either drug, and the PPC score of patients with combined penehyclidine hydrochloride and dexmedetomidine decreased more significantly. However, the application of penehyclidine hydrochloride or dexmedetomidine alone reduced the incidence of PPCs, but not significantly, and combination treatment significantly reduced the incidence of PPCs. We showed that after penehyclidine hydrochloride treatment, the proportion of patients with PPC score 1 increased significantly in the experimental group, while the score in the control group was mainly ≥ 3. These results suggested that penehyclidine hydrochloride or dexmedetomidine reduced the PPC score and severity of postoperative lung injury, but combination treatment reduced the occurrence of PPCs in patients undergoing cardiac surgery. The protective effects of penehyclidine hydrochloride and dexmedetomidine on pulmonary injury have been confirmed in many animal and human studies, but there are few studies on the effects of penehyclidine hydrochloride and dexmedetomidine on pulmonary complications. Dexmedetomidine has been shown to prevent pulmonary complications after oral and maxillofacial reconstruction [24] and video-assisted thoracoscopic surgery [25]. Although the exact protective mechanism remains to be studied, penehyclidine hydrochloride and dexmedetomidine may play a protective role in pulmonary ischemia/reperfusion injury and anti-inflammatory reaction as described previously.

OI is negatively correlated with RI, and these are often used as indicators to evaluate lung function and gas ventilation and exchange. A decrease in OI value indicates a decline in pulmonary ventilation and exchange oxygenation, while a decrease in RI indicates strong pulmonary gas diffusion and exchange capacity and good ventilation volume. In the perioperative period of cardiac surgery with CPB, OI < 300 is usually defined as pulmonary injury [26]. When CPB-related pulmonary injury or ischemia/reperfusion injury occurs, inflammatory cells diffuse and infiltrate the lungs, pulmonary capillary permeability increases, pulmonary tissue is severely edematous, airway and pulmonary vascular resistance increase, and ventilatory insufficiency and hypoxemia occur [27]. Early OI can predict the changes in pulmonary function and extubation time after cardiac valve surgery, and OI and RI are related to the time of ICU mechanical ventilation, and length of ICU and hospital stay [28], proving the sensitivity and accuracy of related respiratory indicators in respiratory management of cardiac surgery. In our study, the control group did not receive penehyclidine hydrochloride and dexmedetomidine. At 30 min after CPB; that is, about 1 h after resumption of pulmonary perfusion and mechanical ventilation, OI decreased significantly compared with before CPB and fell to a level indicating pulmonary injury (OI < 300). At the same time, RI increased quickly after CPB, suggesting that CPB and lung ischemia/reperfusion injury caused acute pulmonary dysfunction after CPB, which is an important cause of PPCs. However, penehyclidine hydrochloride and dexmedetomidine can reduce the decline in OI, which is consistent with penehyclidine hydrochloride reducing inflammatory mediators and improving OI in patients undergoing total arch replacement of aortic dissection [15]. Dexmedetomidine can reduce cell apoptosis, regulate immune factors, and increase OI to play a pulmonary protective role, which has also been confirmed in rat CPB experiments and human studies [29]. Our study showed that combined use of the two drugs improved OI after CPB more significantly than either drug alone, suggesting the synergistic or complementary effect of the two drugs. At 6 h after CPB, OI and RI in each group showed a trend towards improvement, which may have been related to patients with insufficient oxygenation receiving appropriate physical and respiratory therapy in the ICU. Also, the degree of atelectasis was reduced compared with before. Penehyclidine hydrochloride maintained alveolar tension and small airway dilatation, which means that it also maintained a good level of OI at the recovery stage.

Factors such as CPB and surgical trauma can activate systemic inflammatory response syndrome and expand its cascade, which promotes release of proinflammatory cytokines such as TNF-α, leading to pulmonary injury after CPB and serious organ dysfunction after surgery [30, 31]. High levels of TNF-α and IL-6 are closely related to postoperative respiratory dysfunction and prolonged mechanical ventilation [32]. CRP is an acute phase reaction protein originally found in the study of pneumococcal infection, which is used to identify inflammation and severe infection, and guide the treatment of adults and children with antibiotics [33]. IL-6 release stimulates hepatocytes to secrete CRP, which is the main generation mode of CRP [34]. Analysis of clinical data from > 14,000 patients found that the peak concentration of CRP after cardiac surgery was 36 times higher than that before surgery, and the concentration of IL-6 was also high [35]. Procalcitonin is a pro-hormone of calcitonin produced by thyroid C cells and cannot be detected under normal physiological conditions. Some researchers have suggested that procalcitonin is a good predictor of postoperative infection after cardiac surgery [36]. When procalcitonin concentration exceeds the postoperative threshold of 2 ng/ml, the risk of systemic inflammatory response syndrome, cardiopulmonary dysfunction and other complications after coronary artery bypass grafting or valve replacement is increased [37]. Some researchers believe that simultaneous detection of IL-6, CRP and procalcitonin can improve the detection rate of early inflammation in the event of inflammation or infectious diseases [38].

Therefore, prevention and reduction of inflammatory reaction during cardiac surgery with CPB are important for protecting the function of important organs, especially the lungs. Anesthesiologists can reduce the stress damage caused by surgery and CPB to patients by using anesthesia-related drugs and formulating appropriate anesthesia programs. The current study showed that, plasma IL-6 and TNF-α rose rapidly after CPB, reaching a peak at 6 h after CPB, and then decreasing at 24 h, but still significantly higher than preoperatively. This indicated that the inflammatory cascade reaction caused by surgical trauma, CPB, ischemia/reperfusion and other stresses continued into postoperative recovery. These results are consistent with previous animal and human studies in which penehyclidine hydrochloride or dexmedetomidine had an effect on inflammatory factors such as IL-6 and TNF-α [18, 39]. At 6 h after CPB, the concentration of CRP in the four groups began to increase, which was more obvious at 24 h after CPB than preoperatively, which was similar to the typical change trend of CRP after cardiopulmonary bypass [40]. Previous studies found that penehyclidine hydrochloride reduced high-sensitivity CRP in patients with acute ischemia/reperfusion injury after cardiopulmonary resuscitation [41]. Dexmedetomidine, as an α2 agonist, has a potential anti-inflammatory effect and can significantly reduce postoperative CRP [42]. In the current study, penehyclidine hydrochloride or dexmedetomidine alone reduced CRP level compared with the control group, but their combined use did not show a significant advantage compared with single use. This may have been because the observation time in this study was limited to 6 and 24 h after CPB. Compared with IL-6, the change in CRP after CPB shows a slow rising trend [19]. The peak level of CRP may not have been reached by 24 h after CPB, so the full impact of single or combined use of interventional drugs on postoperative level of CRP was not observed. Similarly, the serum procalcitonin concentration in the combination group at 24 h after CPB was lower than that in the control group, indicating a lower risk of inflammation and infection. In our study, the extubation time and length of ICU stay were shortened in the combination group. This supports the above results and analysis of changes in the levels of inflammatory factors, and is consistent with previous studies showing improvement of prognosis by single use of each drug [11].

## Conclusion

The combined use of penehyclidine hydrochloride and dexmedetomidine during anesthesia can inhibit the inflammatory reaction during CPB for cardiac valve surgery, reduce the secretion of inflammatory factors and the occurrence of postoperative pulmonary dysfunction, and improve prognosis of patients.

## Supplementary Information

**Additional file 1: Table A1.** Postoperative pulmonary complications score.

## Data Availability

All data generated and analyzed in the study are available from the corresponding author upon reasonable request.

## Abbreviations

A-aDO_2_: Alveolar arterial oxygen partial pressure difference
ACC: Aortic crossclamp
ACT: Activated clotting time
ALI: Acute lung injury
ASA: American Society of Anesthesiologists
AVR: Aortic valve replacement
BIS: EEG bispectral index
CPB: Cardiopulmonary bypass
CRP: C-reactive protein
DVR: Double valve replacements
FiO_2_: Fraction of inspired oxygen
ICU: Intensive care unit
IL: Interleukin
NYHA: New York Heart Association
OI: Oxygenation index
PaCO_2_: Partial pressure of carbon dioxide in artery
PPCs: Postoperative pulmonary complications
RBC: Red blood cell
RI: Respiratory index
SaO_2_: Arterial oxygen saturation
SpO_2_: Pulse oximetry saturation
TLR: Toll-like receptor
TNF: Tumor necrosis factor
