# Supplementary material for: Effects of penehyclidine hydrochloride combined with dexmedetomidine on pulmonary function in patients undergoing heart valve surgery: a double-blind, randomized trial
Source: BMC Anesthesiol. 2023 Jul 13;23:237. doi: 10.1186/s12871-023-02176-z (PMC10339561; doi:10.1186/s12871-023-02176-z)
Supplement: Supplementary file 1 — Additional file 1: Table A1. Postoperative pulmonary complications score. [file 12871_2023_2176_MOESM1_ESM.docx]

**Appendix**

**Table A1** Postoperative pulmonary complications score

| **Postoperative pulmonary complication score** | |
| --- | --- |
| Grade 1 | Cough, dry  Microatelectasis: abnormal lung findings and temperature > 37.5℃ without other documented cause: normal chest radiograph  Dyspnea, not due to other documented cause |
| Grade 2 | Cough, productive, not due to other documented cause  Bronchospasm: new wheezing or pre-existent wheezing resulting in a change in therapy  Hypoxemia (SpO_2_ ≤ 90%) at room air  Atelectasis: gross radiological confirmation (concordance of 2 independent experts) plus either temperature > 37.5℃ or abnormal lung findings  Hypercarbia (PaCO_2_ > 50 mmHg), requiring treatment |
| Grade 3 | Pleural effusion, resulting in thoracentesis  Pneumonia: radiological evidence (concordance of 2 independent experts) plus clinical symptoms (two of the following: leucocytosis or leucopenia, abnormal temperature, purulent secretions), plus either a pathological organism (by Gram stain or culture), or a required change in antibiotics  Pneumothorax  Noninvasive ventilation, strictly applied to those with all of the following: a) oxygen saturation (SpO_2_) lower than 92% under supplemental oxygen; b) need of supplemental oxygen > 5 L/min; and RR ≥ 30bpm  Re-intubation postoperative or intubation, period of ventilator dependence (non-invasive or invasive ventilation) ≤ 48 hours |
| Grade 4 | Ventilatory failure: postoperative ventilator dependence exceeding 48 hours, or reintubation with subsequent period of ventilator dependence exceeding 48 hours |
| Grade 5 | Death before hospital discharge |
| *We only classified as grade 2 if two or more items in the grade 2 were present. | |
